# DietBet: A Web-Based Program that Uses Social Gaming and Financial Incentives to Promote Weight Loss

**DOI:** 10.2196/games.2987

**Published:** 2014-02-07

**Authors:** Tricia Leahey, Jamie Rosen

**Affiliations:** ^1^Brown Medical SchoolDepartment of Psychiatry and Human BehaviorThe Miriam Hospital's Weight Control and Diabetes Research CenterProvidence, RIUnited States; ^2^DietBet, IncNew York, NYUnited States

**Keywords:** commercial weight loss, Web-based intervention, social gaming, financial incentives

## Abstract

**Background:**

Web-based commercial weight loss programs are increasing in popularity. Despite their significant public health potential, there is limited research on the effectiveness of such programs.

**Objective:**

The objective of our study was to examine weight losses produced by DietBet and explore whether baseline and engagement variables predict weight outcomes.

**Methods:**

DietBet is a social gaming website that uses financial incentives and social influence to promote weight loss. Players bet money and join a game. All players have 4 weeks to lose 4% of their initial body weight. At enrollment, players can choose to share their participation on Facebook. During the game, players interact with one another and report their weight loss on the DietBet platform. At week 4, all players within each game who lose at least 4% of initial body weight are declared winners and split the pool of money bet at the start of the game. Official weigh-in procedures are used to verify weights at the start of the game and at the end.

**Results:**

From December 2012 to July 2013, 39,387 players (84.04% female, 33,101/39,387; mean weight 87.8kg, SD 22.6kg) competed in 1934 games. The average amount bet was US $27 (SD US $22). A total of 65.63% (25,849/39,387) provided a verified weight at the end of the 4-week competition. The average intention-to-treat weight loss was 2.6% (SD 2.3%). Winners (n=17,171) won an average of US $59 (SD US $35) and lost 4.9% (SD 1.0%) of initial body weight, with 30.68% (5268/17,171) losing 5% or more of their initial weight. Betting more money at game entry, sharing on Facebook, completing more weigh-ins, and having more social interactions during the game predicted greater weight loss and greater likelihood of winning (*P*s<.001). In addition, weight loss clustered within games (*P*<.001), suggesting that players influenced each others’ weight outcomes.

**Conclusions:**

DietBet, a social gaming website, reached nearly 40,000 individuals in just 7 months and produced excellent 4-week weight loss results. Given its reach and potential public health impact, future research may consider examining whether a longer program promotes additional weight loss.

## Introduction

### Obesity Prevalence and Treatment Availability

Over 45% of the world population and approximately 66% of American adults are overweight or obese [1,2]. Excess adiposity is associated with serious health risks, including diabetes, cardiovascular disease, and some types of cancer [2,3]. Behavioral weight loss programs consistently yield weight loss of 8 to 10kg, which are associated with significant health improvements, including reduced risk for diabetes [4]. However, behavioral programs are largely university-based and are, therefore, only available to a small portion of overweight and obese individuals in need.

### Accessibility of Internet Commercial Weight Loss Programs

In contrast, commercially available weight loss programs have wide reach, particularly Web-based interventions. In fact, a large percentage of individuals who attempt weight loss report using commercial programs [5], and, in recent years, enrollment in Web-based interventions has increased substantially [6]. The appeal of Internet interventions is likely due to the reduced participant burden associated with frequent in-person visits (eg, time, transportation). Another important benefit of Web-based interventions is their inherent reach; given that Internet access has increased exponentially over the past decade [7], Web-based programs are widely accessible to individuals who may not otherwise have access to clinical weight management interventions. Similarly, such programs are accessible 24-hours a day in a variety of locations (home, work, public libraries). Given their appeal and ability to reach large numbers of overweight and obese individuals in need, commercially available Web-based interventions have significant public health potential.

### Evidence for Commercial Weight Loss Programs

Despite their potential to improve public health, the scientific literature on the effectiveness of Web-based commercial weight loss programs is sparse [5]. Only two programs have been rigorously evaluated in randomized trials: eDiets [8,9] and The Biggest Loser Club [10]. Results of the eDiets trials showed that participants assigned to eDiets achieved significantly less weight loss than those assigned to a self-help condition or an Internet behavioral weight loss program [8,11]. Similarly, while the Biggest Loser Club produced greater weight loss than a no treatment control condition, given that the program was 3 months in length, the weight loss was modest (-2.1kg) [9]. These randomized trials have clear benefits and are essential to demonstrate efficacy. However, an important shortcoming is that, given the nature of rigorous, randomized trials (screenings, run-ins, retention efforts, etc), the results may overinflate true engagement, retention, and weight losses outcomes of commercially available programs. Thus, to complement the clinical trial literature, ecologically valid studies are needed that examine real-world enrollees in naturally occurring Web-based commercial weight loss programs and, thus, ascertain true program engagement, retention, and outcome data. Results from such studies may be used to inform consumer decision making and public policy.

This study examined the effectiveness of DietBet, which is a commercially available Web-based program that uses social gaming and financial incentives to promote weight loss. Upon enrollment, players join a game and enter money into a pool. All players have 4 weeks to lose 4% of their initial body weight. During the game, players report their weight and interact on the DietBet platform. At the end of the game, all players within each game who lose at least 4% in 4 weeks are declared “winners” and split the initial pool of money bet at enrollment. The primary aim of this study was to conduct a naturalistic examination of engagement, retention, and weight loss outcomes in DietBet. Previous findings from financial incentive weight loss trials have shown that the possibility of losing large (vs small) amounts of money for not meeting weight goals motivates better overall weight loss [10]. In addition, more frequent self-weighing and greater social influence for weight loss have been found to be associated with better weight outcomes [12-15]. Given these findings, we explored whether: (1) betting more money at enrollment, (2) completing more weigh-ins, and (3) having greater social engagement/influence predict greater percent weight loss and greater likelihood of winning. Finally, given evidence that weight loss clusters in social networks and that group characteristics impact weight outcomes in group-based weight loss competitions [12], we also explored whether weight loss clusters within games (ie, players in the same game achieve similar weight loss) and whether game characteristics (eg, number of players) are associated with weight outcomes.

## Methods

### Procedures

DietBet is a social gaming website that uses financial incentives and social influence to promote weight loss. Players are recruited via press coverage (eg, Today Show, CNN, New York Times, Wall Street Journal), business development efforts (popular wellness experts with social capital are asked to host games and encourage fans/followers to participate), direct virality (players recruit other players), and indirect virality (players share DietBet information on Facebook). At enrollment, players bet money and join a game. Players are given the option to join an existing game that has not yet started or create their own game. If they create their own game, it can be either open (anyone can join) or closed (invite only; ie, all players know each other).

**Figure 1:**
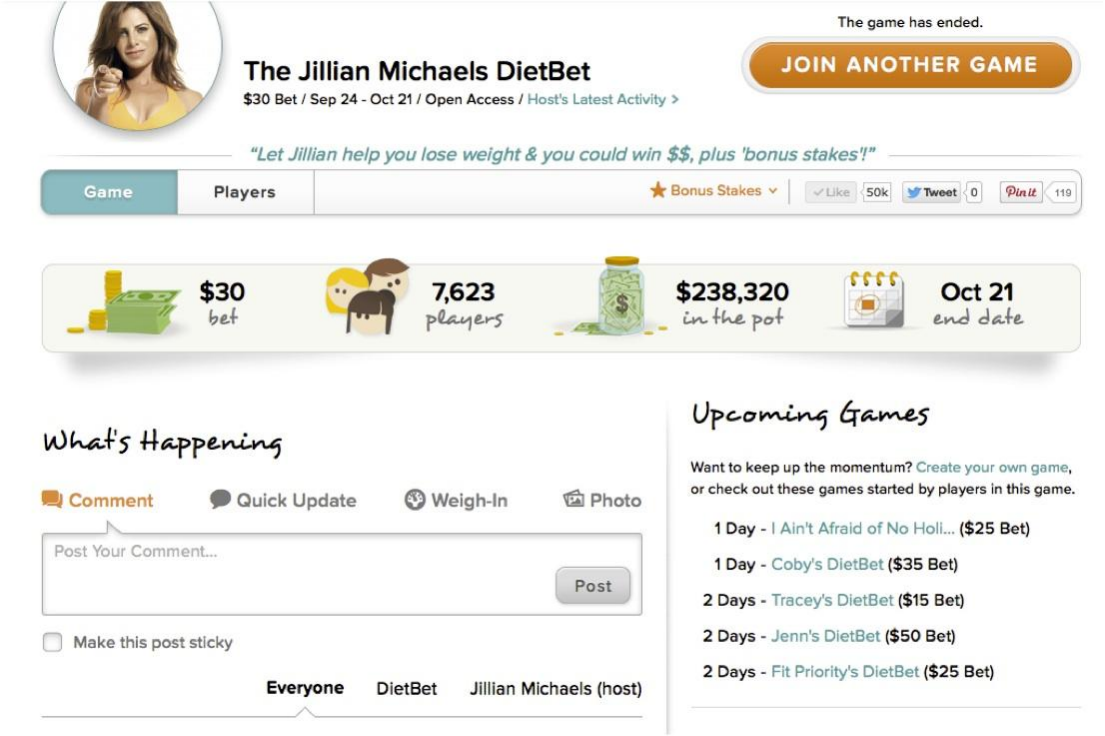
Intervention screenshot.

Players are prompted to submit their official start weight two days prior to the start of the game, (see below for weigh-in procedures). Once the game begins, players have 28 days to lose 4% of their initial body weight. DietBet does not promote a specific diet or weight loss strategy; instead, players are allowed to choose any strategy to lose weight (eg, low-fat diets, low-carbohydrate diets, etc). During the game, players post photos, comments, and weight loss tips (Figure 1 shows a screenshot). They are also encouraged to stay accountable to one another by posting their weight loss. Players can view their weight loss relative to the weight loss of others in the game (see Multimedia Appendix 1). To facilitate game communication and sharing, players have access to the DietBet app, which is designed for all smartphone mobile devices and includes all aspects of the gaming platform. At the end of 28 days, players have 48 hours to send in their final weight. All players who lose at least 4% of their initial body weight in 28 days split the pool of money that was bet at the start of the game. For example, if the game consisted of 10 players who each bet US $25 and 4 people won, after DietBet’s cut (see below), they would each win US $50. If no one loses 4% of their starting weight, the player who lost the most weight wins the pool of money. Winners are notified by email of their payout, which they can either apply to their next DietBet game or cash out.

DietBet keeps a portion of the initial pool of money to cover financial transaction costs and staff time associated with weight verification, customer service, and tech support. DietBet’s share is taken out of each game’s starting pool of money before winners are paid. Thus, players who do not win do not incur a charge to play. DietBet retains 15%-25% of the payout depending on the amount of each individual bet (US $1-$99–25% fee; US $100-$249–20% fee; US $250 or more–15% fee).

### Measures

#### Gender and Weight

Participants report their gender at the beginning of the game. Within 48 hours prior to the start of each game, participants are required to complete an official weigh-in. Similarly, within 48 hours after the end of a game, participants are to complete an official weigh-in. Official weigh-in procedures involve the submission of two photos, one of the player on a scale in light indoor clothing without shoes and another that captures the scale’s readout and a piece of paper that includes the player’s weigh-in password. DietBet staff reviews each photograph for player-password correspondence. In addition, DietBet uses prespecified algorithms to detect any unusual weight outcomes and game activity; specifically, across games DietBet will identify players who have won more than US $300 in DietBets and identify players who have unusual weight gain between DietBet games (ie, gained more than 1% per day). Those individuals are flagged and DietBet staff completes a detailed review of their weight, game activity, and profile information. Using these algorithms and the photo-based system within 48 hours of the start and end of a game, if a weight is deemed questionable, players are required to submit extra proof by completing a live Skype weigh-in with one of DietBet's referees, a video weigh-in, or an in-person weigh-in at a DietBet approved location (eg, Walgreens drugstores or Customer Value and Service drugstores).

#### Weigh-Ins, Social Engagement, and Facebook Shares

All weigh-in, social engagement, and Facebook data were obtained directly from the DietBet website. Specifically, during the game, players reported their weight on the DietBet platform and weigh-ins were summed for each player. Players interacted with one another by cheering, posting pictures, posting status updates, “liking” others’ posts, and commenting on and replying to posts; to create an overall social engagement variable, the number of social interactions for each variable (cheers, pictures, updates, etc) were summed for each player. DietBet collected data on whether players posted their game participation on Facebook; data were coded 1 for “Yes” or 0 for “No.” The option to share on Facebook was not available in early games. Thus, the sample size for this variable is smaller than the overall sample (n=20,059 vs N=39,387).

#### Money Bet and Won

The amount of money bet and the amount of money won was obtained objectively via payment transaction data.

#### Game Characteristics

The number of players on each game and whether all players knew each other (closed game/invite only vs open game) were obtained objectively by extracting data from the platform.

#### Statistical Analyses

Player characteristics, engagement, and completion outcomes were examined using simple descriptive statistics, including means, standard deviations, and percentages. Completers (ie, those who completed an official weigh-in procedure at the end of the game) and noncompleters were compared using analyses of variance or chi-square tests for continuous or categorical variables, respectively. To examine the effects of DietBet on weight loss, a conservative baseline carried forward intention-to-treat approach was used; players who did not finish the game (ie, did not complete a final, verified weigh-in) were assumed to have remained at baseline/entry weight and within subjects *t* tests were conducted. Predictors of weight loss and whether a player won their game were examined with regression analyses. In addition, to determine which variables explained unique variance in weight loss, a multivariate analysis that included all variables was conducted. The effects of game and game characteristics on weight outcomes were also explored. To examine whether weight loss clustered within games, an unconditional multilevel model was conducted and an intraclass correlation coefficient (ICC) was calculated using the resulting between and within group variance components, (ICC=U_0_/U_0_+R).

#### Ethics

The Miriam Hospital’s Institutional review board approved this study.

## Results

### Engagement and Completion

From December 2012 to July 2013, 39,387 players participated in 1934 games on the DietBet platform. Players were predominantly female (84.04%, 33,101/39,387) with a mean baseline weight of 87.8kg (SD 22.6kg). The average amount of money bet at game entry was US $26.84 (SD US $21.93). Upon enrolling, 50.03% of players chose to share their DietBet participation on Facebook (note–the option to share on Facebook was not available in earlier games, thus the total sample size for this variable was n=20,059, of those n=10,036 shared their DietBet participation). During the 4-week game, players completed an average of 5.3 (SD 3.9) weigh-ins and engaged in 9.3 (SD 78.4) social interactions (eg, cheers, posts, likes, etc).

A total of 71.71% of participants (28,244/39,387) self-reported their weight into the DietBet platform during week 4, and 65.63% (25,849/39,387) completed an official weigh-in at the end of the game (ie, completed the photo-based weigh-in process immediately following the game). A greater proportion of men completed a photo-verified weigh-in than women (Men–68.39%, 4292 out of 6275; Women–65.10%, 21,550 out of 33,101; *P*<.001). Compared to noncompleters, completers weighed less at baseline (87.4kg, SD 22.2kg vs 88.5kg, SD 23.3kg, *P*<.001), bet more money at program entry (US $27.79, SD US $24.99 vs US $25.01, SD US $14.21, *P*<.001), and had more weigh-ins (6.5, SD 4.0 vs 3.0, SD 2.5, *P*<.001) and more social interactions with their teammates during the game (eg, cheers, comments, likes, etc; 12.0, SD 95.3 vs 4.3, SD 22.6, *P*<.001). A greater percentage of completers versus noncompleters shared their game participation on Facebook (50.96%, 7074 out of 13,882 completers vs 47.74%, 2949 out of 6177; *P*<.001).

### Weight Loss

Intention-to-treat analyses (assuming that individuals who did not complete an official, photo-based weigh-in at the end of the game did not lose any weight) showed that players lost a significant amount of weight from baseline to the end of the game (*P*<.001; Table 1). The average weight loss was 2.6% (SD 2.3%). Out of the 39,387 players who enrolled, 43.60% (n=17,171) were winners (ie, lost at least 4% of initial body weight or, if no one lost 4%, lost the most weight in their game). The average amount won was US $58.79 (SD US $34.90) (net earnings–US $29.00, SD US $16.43). Game winners lost an average of 4.9% (SD 1.0%) of initial body weight. A total of 30.68% of winners (n=5268) achieved a 5%, or clinically meaningful, weight loss.

**Table 1:** Player weight loss.

	Total sample (N=39,387)	All winners (n=17,171)	Winners who lost ≥ 4% (n=16,687)	Winners who lost most weight in game (n=484)
% weight loss, mean (SD)	2.6 (2.3)	4.9 (1.0)	4.9 (0.9)	3.4 (0.9)

### Participant Characteristics Associated With Weight Loss and Winning the Game

Participant baseline characteristics and game variables predicted weight loss outcomes and whether a player won their game. Significant predictors of greater percent weight loss were male gender (*P*<.001), lower baseline weight (*P*=.03), betting more money at game entry (*P*<.001), completing more weigh-ins during the game (*P*<.001), sharing game participation on Facebook (*P*<.001), and having more social interactions with other players (*P*<.001). These same variables were also significant predictors of whether a player won their game (*P*s<.001; Table 2).

**Table 2:** Winners’ versus nonwinners’ baseline characteristics and engagement.^a^

	Winners (n=17,171)	Nonwinners (n=8678)	Winners versus nonwinners (*P* value)
Male, n (%)	3489 (20.32)	803 (9.25)	<.001
Baseline weight kg, mean (SD)	87.1 (22.1)	88.1 (22.4)	<.001
Amount bet US $, mean (SD)	$29.79 ($29.18)	$23.83 ($12.30)	<.001
Shared on Facebook,^b^ n (%)	4837 (53.19)	2241 (46.79)	<.001
Weigh-ins, mean (SD)	7.0 (4.2)	5.4 (3.1)	<.001
Social engagement, ^c^ mean (SD)	13.6 (113.6)	8.8 (38.4)	<.001

^a^Given that completion was associated with baseline characteristics and engagement, to control for potential confounding of completion, only game completers were included in these analyses, n=25,849.

^b^Option to share on Facebook was not available in early games. Thus, the sample size for this variable is smaller than the overall sample of completers. Specifically, of those who were given the option to share on Facebook, n=20,059, a total of n=13,882 completed the game. Of those, n=9093 were winners and n=4789 were nonwinners.

^c^Sum of cheers, comments, replies, likes, photos, and updates.

In a multivariate model, all variables remained significant, independent predictors of weight loss (*P*s<.001) with the exception of number of social interactions and whether a player shared on Facebook (*P*s>.38).

### Game Characteristics and Social Network Factors Associated With Weight Loss

The 39,387 players represented a total of 1934 games. Weight loss tended to cluster within games (*P*<.001; ICC=.07, consistent with a small effect) [16], suggesting that a player’s weight loss was influenced by other players’ weight loss. On average, there were 31.4 (SD 171.2) players in each game. Game size predicted weight loss; larger games were associated with greater weight loss (*P*<.001). Games in which players knew one another had slightly more social interactions than games in which players did not know one another (ie, invite only/closed games vs open games; 4.9, SD 6.1 vs 4.1, SD 5.1, *P*=.001); however, weight loss outcomes were not affected by whether players knew one another at the start of a game (*P*=.74).

## Discussion

### Summary of DietBet Engagement and Weight Loss

DietBet, a 4-week commercial Web-based program for weight loss, yields retention results that are comparable to other programs [9,17] and produces excellent engagement and weight loss. On average, participants interacted with other players on the DietBet platform more than twice a week and reported their weight loss at least once a week. A total of 71.71% of participants (28,244/39,387) submitted a self-reported weight at week 4 and 65.63% (25,849/39,387) completed final, photo-based weigh-in verification procedures. Average intention-to-treat weight loss was 2.6%, and 42.39% of players (16,696 out of 39,387) achieved the 4% weight loss goal. Moreover, over 5000 participants (n=5268) achieved a 5%, or clinically meaningful, weight loss.

### Comparison of DietBet Results to Other Internet Commericial Weight Loss Programs

DietBet results compare favorably to other Web-based commercial programs and to more intensive programs. In reports of the Biggest Loser Club, participants self-reported their weight loss less than once a week [18], social engagement was low (median interactions=0, range 0-0) [18], and overall weight loss ranged from 2% to 3% [9,18]. Similarly, reports on the eDiets program indicate low engagement and modest weight loss (1% to 3%), even when combined with in-person visits and when evaluated in a randomized trial that would presumably improve adherence and outcomes [8,11]. Given that the Biggest Loser Club and eDiets are longer programs (3- and 6-months, respectively) and that weight loss steadily increases during the initial 4 to 6 months of treatment [19], these earlier programs would be expected to yield a greater magnitude of weight loss than DietBet. Instead the one month weight losses produced by DietBet are as good as, if not better than, those produced by 3- and 6-month programs. In fact, they are comparable to weight losses achieved during the initial four weeks of university-based, intensive behavioral weight loss programs that involve weekly in-person meetings led by professional staff [19]. While these results are promising, in order to draw more definitive conclusions regarding the weight loss produced by DietBet versus other commerical weight loss programs, the length of DietBet needs to be extended so that it is more consistent with the length of these longer, more intensive commerical weight loss interventions.

### Explaining DietBet Results: Behavioral Economics, Social Influence, and Self-Monitoring

The favorable outcomes produced by DietBet may be attributed to its social gaming components-namely, the use of financial incentives and social influence. Results showed that those who bet more money and had greater social engagement had a greater magnitude of weight loss and were more likely to “win” their game. These findings are consistent with those from behavioral economics and with findings in the behavioral weight loss literature. Behavioral economics suggests that loss aversion (the strong tendency to avoid losing something that is owned) is a significant motivator of human behavior and that the magnitude of loss may moderate the effect, with greater potential loss yielding greater motivation [20]. Consistent with this theory, in several randomized trials Jeffery et al showed that behavioral weight loss programs involving deposit contracts (participants deposited money and got it back for meeting goals) yielded significantly greater weight loss relative to the same behavioral interventions without such contracts [21,22]. Moreover, participants who deposited more money at baseline, and could have therefore lost more money, were more likely to reach weight loss goals [22]. There is also strong evidence that combining financial incentives with social influence further improves outcomes. Specifically, randomized trials have shown that group incentives for meeting weight goals, either collaborative or competitive, are more effective than individual incentives [14,21,23]. Thus, the excellent weight losses, retention, and engagement produced by DietBet are likely due to its use of principles from behavioral economics and its inclusion of financial incentive and social influence strategies. Given this success, future Internet interventions, commercial or otherwise, may consider harnessing financial incentives and social influence for weight loss to promote optimal outcomes.

Players who reported their weight loss more often also lost more weight. Not only is regular reporting indicative of better program engagement, which alone is associated with better outcomes [13], but regular weighing is also linked to better weight loss results [24], likely via the process of self-regulation [25]. Specifically, getting on the scale on a regular basis yields important information on whether weight loss efforts are working and, if not, communicates the need to reduce dietary intake and increase physical activity to reach weight goals. Thus, consistent with intervention recommendations from university-based behavioral weight loss programs, DietBet players weighed themselves an average of at least once a week, which likely contributed to the positive weight loss results achieved.

In addition to examining individual effects, we also explored the effects of game characteristics on outcomes. Consistent with previous findings showing that weight loss clusters in team-based weight loss competitions [12], individuals in the same game tended to achieve similar weight losses, suggesting social influence for weight loss among players. In addition, games with more players achieved greater weight loss overall. While previous research shows that group size does not affect weight loss outcomes in group-based interventions [12], this earlier study did not involve incentives. However, this earlier study did not involve incentives. Consistent with the behavior economics principle that reinforcement size is positively associated with response strength [26], the larger pool of money inherent in bigger games may have motivated players to lose more weight to, thereby, “win” their game. Interestingly, player familiarity created by “invite only” games (vs open games) did not affect weight outcomes. Combined with the clustering effect, these results suggest that games are able to effectively create social influence and promote social interactions for weight loss even among strangers.

### Study Limitations

Study limitations include a predominantly female sample, lack of some player characteristic data (age, race, ethnicity, etc), the short program length, and lack of fully objective weight data. Majority female participation is common in weight loss trials and commercial weight loss programs [8,9,18]; however, given the prevalence of obesity in men [2], future DietBet games may consider using targeted advertisements to increase male enrollment. Additional player information (eg, height, age, race, ethnicity) would help to better describe the large sample of individuals who enrolled in DietBet and make comparisons between the DietBet population and populations of other Internet commercial weight loss programs; DietBet has begun to collect such information on new enrollees. The program length was 4 weeks; while players achieved excellent weight losses in this short period of time, and there is evidence that initial weight loss is predictive of future success [27], a longer program is warranted. As such, DietBet has developed and launched a longer, 6-month game, which will allow us to examine magnitude of weight loss produced over a longer timeframe. Finally, while the DietBet weigh-in system is more rigorous than the self-report methods used in other commercial programs [19], a systematic validation study comparing the DietBet photo-based weigh-in system to objective weights obtained from unbiased assessors is warranted.

### Study Strengths

This study has several strengths. It provides a reliable and ecologically valid examination of the true engagement, retention, and weight loss achieved in DietBet, a wide-reaching, commercial, Web-based weight loss program that uses financial incentives and social influence to promote weight loss in large numbers of individuals. Only a small number of studies have conducted a naturalistic examination of such programs. Moreover, these previous studies have obtained only self-report weight [17,18,28]. In contrast, the weigh-in procedures used in DietBet were not solely reliant on self-report; instead, players were required to provide photo-based weight information and rigorous weight verification procedures were employed, with human referees reviewing multiple photos per player. Moreover, as indicated by the Federal Trade Commission, the data provided herein are critical to consumers who are inundated with Web-based weight loss options, but have limited information on effectiveness [29]. Finally, and perhaps most importantly, given that DietBet was able to reach nearly 40,000 individuals in just 7 months and, with the use of financial incentives and social influence, engage players and produce promising weight losses, DietBet may have the potential to impact public health and help address the epidemic of obesity.

## Multimedia Appendix 1

Intervention screenshot of player progress.

## Abbreviations

ICC: intraclass correlation coefficient
