# Supplementary figures and images for: DietBet: A Web-Based Program that Uses Social Gaming and Financial Incentives to Promote Weight Loss
Source: JMIR Serious Games. 2014 Feb 7;2(1):e2. doi: 10.2196/games.2987 (PMC4307813; doi:10.2196/games.2987)

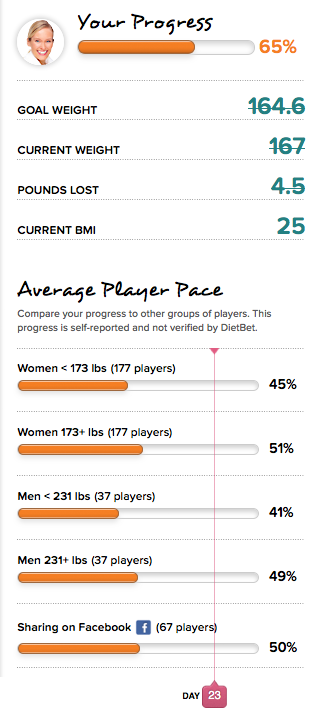

Supplement: Supplementary file 1 [file games_v2i1e2_app1.png]
